# Supplementation with sesame oil suppresses genotoxicity, hepatotoxicity and enterotoxicity induced by sodium arsenite in rats

**DOI:** 10.1186/s12944-022-01760-5

**Published:** 2023-01-27

**Authors:** Akinleye Stephen Akinrinde, Stephen Oluwasemilore Oyewole, Olufunke Eunice Ola-Davies

**Affiliations:** Department of Veterinary Physiology and Biochemistry, Faculty of Veterinary Medicine, University of Ibadan, Ibadan, Nigeria

**Keywords:** Sodium arsenite, cytotoxicity, mucin, apoptosis, colon, liver, sesame

## Abstract

**Background:**

Sesame oil, an edible essential oil, is known to be rich in unsaturated fatty acids, vitamins and lignans with several reported health-promoting benefits. Acute arsenic poisoning produces toxic hepatitis, bone marrow depression and adverse gastrointestinal responses. In this study, we investigated the protective effect of sesame seed oil (SSO) against genotoxicity, hepatotoxicity and colonic toxicity induced by sodium arsenite (SA) in Wistar rats.

**Methods:**

Twenty-eight male Wistar albino rats were randomly allocated into four groups: control, SA only (2.5 mg/kg), SA + SSO (4 ml/kg) and SSO alone for eight consecutive days. Liver function and morphology, bone marrow micronuclei induction, colonic histopathology, mucus production and immune expression of Bcl-2, carcinoembryonic antigen (CEA), MUC1 and cytokeratins AE1/AE3 were evaluated.

**Results:**

SA provoked increased serum activities of liver enzymes, including alanine aminotransferase (ALT) and aspartate aminotransferase (AST), and caused severely altered morphology of hepatic and colonic tissues with increased frequency of micronucleated polychromatic erythrocytes (MnPCEs/1000PCE) in the bone marrow. In addition, SA triggered increased expression of colonic CEA and MUC1 but weak Bcl-2 immunoexpression. However, cotreatment with SSO demonstrated protective activities against SA-induced damage, as indicated by significantly reduced serum ALT and AST, fewer micronucleated bone marrow erythrocytes and well-preserved hepatic and colonic morphologies compared to the SA-treated rats. Furthermore, SSO protected the colonic mucosa by boosting mucus production, elevating anti-apoptotic Bcl-2 expression and reducing CEA expression. GC–MS analysis of SSO revealed that it was predominated by linoleic acid, an omega-3 fatty acid, and tocopherols.

**Conclusions:**

Our data indicated that SSO protected the liver, colon and bone marrow potentially via anti-inflammatory and anti-apoptotic activities. The data suggest that sesame oil has potential therapeutic applications against chemical toxicities induced by arsenic.

## Introduction

Arsenic toxicity continues to be an important occupational and environmental health problem arising from exposure to arsenic compounds via contamination of groundwater and food meant for human consumption from industrial activities and agrochemical wastes such as arsenical pesticides, herbicides or rodenticides [1]. Ingested arsenic provokes acute symptoms of acute gastrointestinal irritation, such as nausea, vomiting and profuse diarrhea, while its eventual absorption and distribution to other organs, such as the lungs, liver and kidneys, result in a wide variety of clinical symptoms, including circulatory insufficiency, cardiomyopathies, and hepatic and renal failure [2]. Long-term exposure to arsenic has been associated with different types of cancer, especially skin cancer, immunotoxicity, and endocrine and nervous dysfunctions [3].

The main cellular mechanisms of arsenic toxicity include induction of mutations and chromosomal aberrations, alteration of signal transduction, cellular differentiation and apoptosis, alterations in gene expression and induction of oxidative stress [4]. Various studies have provided evidence for the important role of oxidative stress in arsenic toxicity through excessive production of reactive oxygen species (ROS), reduction in antioxidant capacity via reduction of cellular glutathione and inhibition of the activity of antioxidant enzymes [5, 6]. Arsenic compounds have also been widely investigated for their ability to induce genotoxicity and chromosomal abnormalities, which, along with the ability to induce epigenetic changes, are thought to be responsible for the ability of these compounds to induce tumorigenesis. The clastogenic effects of arsenic are thought to be due to its high affinity for sulfhydryl groups of proteins associated with DNA [7].

Organs such as the liver and kidneys are considered to be important target organs of arsenic toxicity due to their roles in xenobiotic metabolism and excretion of metabolites, respectively [8]. However, studies on the intestinal effects of arsenic are still very few. As the first barrier against oral exposure to toxic chemicals in the diet, the gastrointestinal tract must be considered a critical target for arsenic toxicity, as the integrity and responses of these tissues may be important in modulating the absorption and toxicity of this chemical to other internal organs [9, 10]. Earlier reports revealed histological alterations such as rupture and degeneration of the intestinal villi and the gastric mucosa in rats treated with sodium arsenite [11], while other studies reported lesions including pronounced hyperplasia of the crypts and cells lining the mucosal glands, erosions and ulcerations [12]. Compared to other segments of the gastrointestinal tract, the colonic epithelium is likely to be more susceptible to the putative toxic effects of arsenic because of the relatively longer residence time (average of 1-2 days) of feces in this segment, potentially offering more opportunity for interaction with arsenic and its metabolites [13].

Recently, several plant products and their active constituents have received great attention as potential protective agents against arsenic-induced toxicity. Sesame oil is an edible vegetable oil obtained from the seeds of the sesame plant *Sesamum spp.* and it grows throughout the tropical and subtropical areas of Asia, Africa, and South America. Like most edible oils, sesame seed oil (SSO) consists mainly of acyl lipids (triacyglycerols) and fatty acids such as palmitic, stearic, oleic and linoleic acids. There has been a considerable increase in consumer interest in the consumption of essential fatty acids, such as oleic acid (OA) and α-linoleic acid (ALA), which are now recognized as functional foods due to their various health benefits. For instance, ALA is well known for its anti-inflammatory activities, which are believed to be mediated by its ability to interfere with arachidonic acid metabolism, thereby inhibiting the biosynthesis of pro-inflammatory prostaglandins [14]. Thus, diets rich in ALA have been shown to ameliorate inflammatory states in the intestines [15] and cardiovascular system [16].

The nonacyl component of sesame oil constitutes a much lesser fraction and is made up primarily of two major lignans, sesamin and sesamolin, along with small amounts of other phenolic compounds such as sesaminol, sesamolinol, pinoresinol, and larisiresinol, which are reportedly responsible for the antioxidant activity of SSO [17, 18]. In addition to these bioactive compounds, sesame oil is also a rich source of total tocopherols, predominantly γ-tocopherol and small amounts of δ-tocopherol, α-tocopherol and β-tocopherol [19]. Various health-promoting effects, such as antioxidant, antihypertensive, antiproliferative, and neuroprotective activities, as well as cholesterol-lowering properties, have been associated with SSO, and these are thought to emanate from its bioactive chemical constituents, especially sesamin and sesamolin [20, 21]. However, the effects of SSO on arsenic-induced damage to the liver, intestines and bone marrow have not been investigated. The present study has therefore been carried out to understand the protective role of SSO on hepatotoxicity, genotoxicity in bone marrow tissues and colonic toxicities induced by sodium arsenite administration in rats.

## Materials and methods

### Chemicals

Sodium arsenite (SA) was purchased from Loba Chemie Pvt Ltd. (Mumbai, India) (0577500025; Lot # A167051603). Sesame seed oil (SSO) was acquired from Hemani Herbal LLC (Longwood, FL, USA). Colorimetric kits for alanine aminotransferase (ALT), aspartate aminotransferase (AST) and alkaline phosphatase (ALP) assays were obtained from Randox® Laboratories (Randox® Laboratories Ltd, Ardmore, United Kingdom). Anti-MUC1, anti-Bcl-2, anti-CEA and anti-AEI/AE3 antibodies were obtained from Dako North America Inc. (Real Carpinteria, CA, USA). The doses of SA and SSO used in this study were selected based on findings from previous studies [22, 23].

### Gas chromatography–mass spectrometry (GC–MS) analysis of SSO

GC–MS analysis of SSO was carried out using an Agilent 6890 gas chromatography system coupled with an Agilent 5975 mass detector (Chemetrix, Pty, Ltd, Agilent Technologies, DE, Germany). The protocols were as described previously by Akinrinde *et al.* [24]. Briefly, samples were injected as 1 mL splitless injections into the GC column (a Zebron-5MS column, 30 mm × 0.25 mm × 0.25 μm) (Phenomenex Inc. Torrance, CA, USA) consisting of cross-linked 5% phenylmethylpolysiloxane. GC-grade helium was used as the carrier gas and was maintained at a flow rate of 2 mL/min with column temperature ranging between 70 and 270°C, ramped at intervals of 15-20°C/min. Data were acquired with ChemStation Integrator, and chemical constituents of the oil were identified based on computer matching with mass spectra stored in the installed NIST11.L library [25]. The relative percentage of oil constituents was calculated from the peak areas.

### Animals and treatments

Twenty-eight (28) male Wistar rats (200 ± 10 g) were obtained from the animal house of the Experimental Animal Unit, Faculty of Veterinary Medicine, University of Ibadan, Nigeria. They were housed in plastic cages under standard conditions with a room temperature of approximately 22 ± 2°C and a 12 h dark/12-h light photoperiod cycle, with free access to standard rat chow and clean tap water. The experiments were conducted according to guidelines outlined in the National Institute of Health publication, “Guide for the Care and Use of Laboratory Animals” [26] and followed guidelines approved by the Animal Care and Use Research Ethics Committee of the University of Ibadan (UI-ACUREC File No.: 22/013).

The rats were randomly allocated into four experimental groups (A-D) consisting of seven rats each. Group A (control group) received normal saline; Group B received SA (2.5 mg/kg/day) orally for 8 days; Group C was treated concurrently with SA and concurrently with SSO (4 mL/kg/day) orally for 8 days. Group D was treated with SSO (4 mL/kg/day) only for 8 days.

### Blood and tissue collection

Approximately 24 h after the last administration, blood was collected from the retro-orbital venous plexus of the rats into plain sample bottles under xylazine/ketamine (5 mg/kg/40 mg/kg) anesthesia. Rats were then euthanized by cervical dislocation, after which the femur bones, liver and colon tissues were isolated and rinsed with normal saline. Blood samples were centrifuged at 3000 rpm for 10 min to obtain the serum as supernatant. The bone marrow was processed for the assessment of genotoxicity, while the liver and colon tissues were immediately fixed in 10% phosphate buffered formalin and were later processed for histological and immunohistochemical assessments.

### Micronucleus Assay

The clastogenic effect of SA was evaluated using the micronucleus assay as described by Heddle and Salamone [27]. Bone marrow from both femur bones in each rat was flushed with fetal calf serum (FBS) on glass slides. The marrow suspension was positioned and smeared on one end of a slide at an angle of approximately 45 degrees. The preparations were air-dried for 24 h. Slides were then fixed in methanol for 5 minutes, allowed to dry and later stained with May-Gruenwald and Giemsa stains. Slides were thereafter rinsed in phosphate buffer and in distilled water, air-dried and mounted in DPX with cover slips. They were later viewed and scored with a light microscope to estimate the frequency of micronucleated polychromatic erythrocytes (MnPCE)/1000 PCE. The assessments were carried out by an independent observer who was blinded to the treatments.

### Assay of serum biochemical parameters

The activities of alanine aminotransferase (ALT), aspartate aminotransferase (AST) and alkaline phosphatase (ALP) were determined using colorimetric assay protocols [28] as described in commercial Randox® kits (Randox Laboratories, Ltd, Ardmore, UK).

### Histopathology

Small pieces of liver and colon tissues initially fixed in phosphate buffered formalin were dehydrated in graded ethanol concentrations and then embedded in paraffin, after which sections (4-5 μm) were made. The sections were later affixed on plain glass slides as duplicates. One set of slides was stained with hematoxylin and eosin (H&E) for the examination of general lesions [29], while the other set was stained with periodic acid-Schiff (PAS) for the demonstration of mucus glycoproteins [30]. Approximately 10-15 microscopic fields were examined using a light microscope by an independent pathologist who was blinded to the treatments.

### Immunohistochemistry

Immunohistochemistry protocols were carried out on formalin-fixed, paraffin-embedded colon sections to demonstrate the expression of Bcl-2, CEA, MUC-1, and cytokeratin AE1/AE3, according to methods described by Todorich *et al.* [31]. Briefly, slides were deparaffinized and rehydrated using xylene and graded alcohol concentrations (100% - 75%). For antigen retrieval (i.e., unmasking of antigenic epitopes), the slides were immersed in approximately 250-300 mL preheated citrate buffer (10 mM; pH 6.0)/EDTA buffer (pH 9.0) inside a coupling jar and incubated in a water bath for 10-20 min. The slides were allowed to cool and then rinsed in wash buffer. Thereafter, 10% fetal bovine serum in phosphate buffered saline was added onto the slides as a blocking agent to prevent nonspecific antigen binding. Afterwards, 130 μL of appropriately diluted anti-Bcl-2, anti-CEA, anti-MUC-1 and anti-AE1/AE3 primary antibodies was added to the sections on the slides and then incubated in a humidified chamber at room temperature for 1 h. Then, 130 μL of appropriately diluted antibody amplifier + Polymer-HRP Micropolymeric –HRP secondary antibody was applied to the slides and incubated for 15 min, following which 130 μL of DAB solution (freshly made before use) was applied to the slides. The slides were then counterstained in hematoxylin and later dehydrated by incubation in alcohol (95%, 95%, 100% and 100%) for 5 min each. Xylene was then applied for clearing, and cover slips were applied along with a mounting solution. Immunostaining regions in the tissue sections were then observed using a light microscope, and a digital camera was used to obtain photomicrographs.

### Statistical Analyses

The results were analyzed with GraphPad Prism software (version 7.00) and are expressed as the mean±standard deviation. Statistical significance was tested by one-way analysis of variance (ANOVA) followed by Tukey’s *post hoc* test for multiple comparisons. *P* values less than 0.05 were considered statistically significant. For semiquantification of immunohistochemical staining, approximately 10-15 microscopic fields from each group were subjected to analysis on Fiji (ImageJ) software (version 1.53c, National Institutes of Health, USA), according to methods described by Crowe and Yue [32] for the semiquantitative determination of protein expression using immunohistochemistry staining.

## Results

### Chemical composition of SSO

**Fig. 1:**
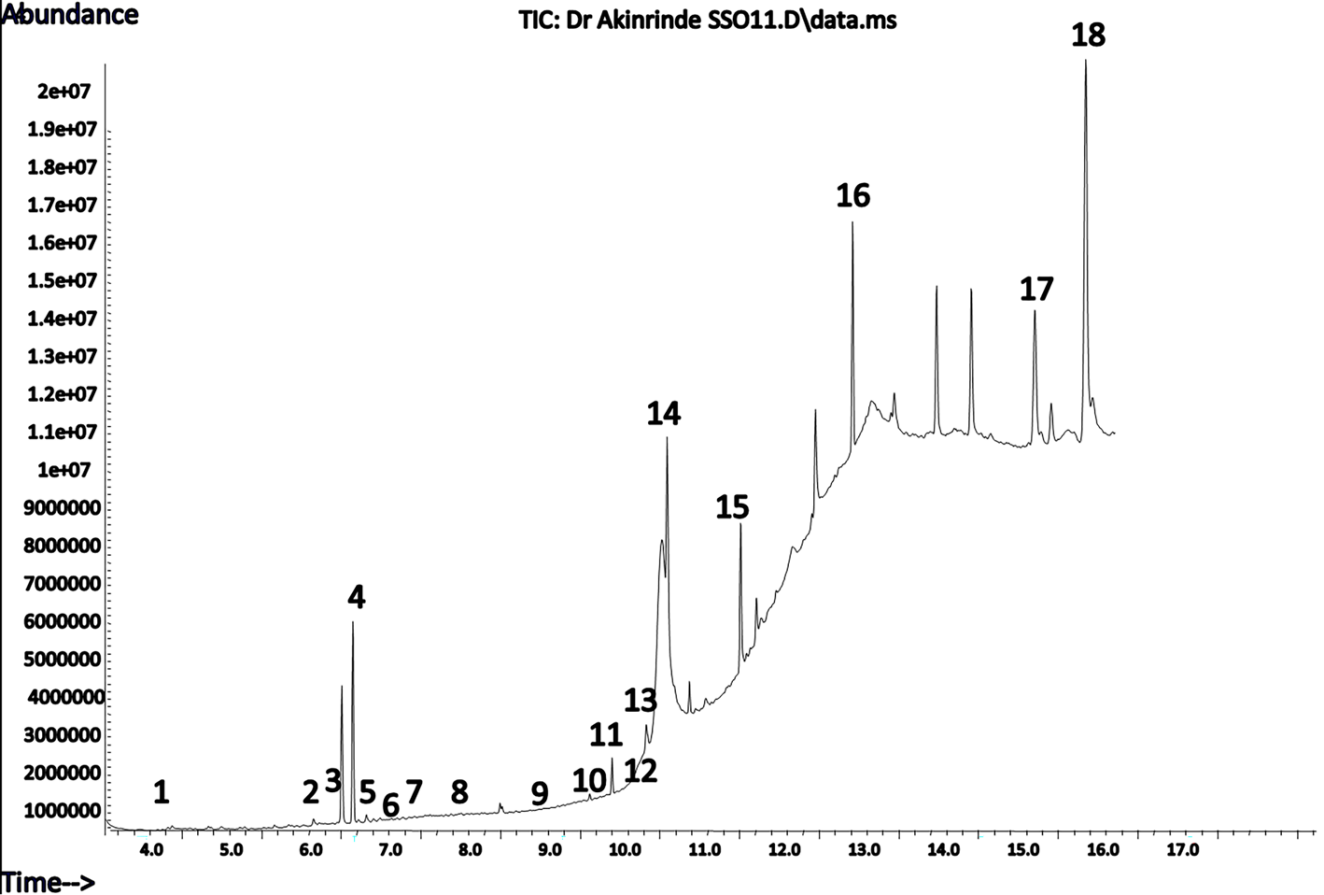
GC–MS chromatogram of sesame oil. 1. 10-Bromo undecanoic acid; 2. Cholestane-3,7,12,25-tetrol; 3. Benz (a) anthracene -7-carbonitrile; 4. (E,E)-2,4-Decadienal; 5. Phthalic acid, di-(3,5-dimethylphenyl) ester; 6. 1,2-Dihydropyrido (3,2,1-kl)phenothiazin-3-one; 7. Methyl 2-oxo octadecanoate; 8. 1,5-Diphenyl-1H-1,2,4-triazole-3-thiol; 9. 6-Carboxypterin; 10. 1-(1-Adamantyl carbonyl)-4-(2-furoyl) piperazine; 11. N,N-Dimethyl-1-adamantyl carboxamide; 12. β-alanyl-L-alanine; 13. Hexadecanoic acid methyl ester; 14. α-linoleic acid; 15. Linoleic acid ethyl ester; 16. Butyl octadeca-9,12-dienoate; 17. γ-Tocopherol; 18. DL-α-Tocopherol

Due to the variability in composition, properties and bioactivities of sesame oils extracted from different locations and by varying methods, we decided to subject the commercially obtained oil to chemical analysis by GC–MS to characterize its bioactive constituents. The analysis revealed a total of eighteen identified chemical constituents, representing 94.56% of the total oil content. The percentage composition of SSO is presented in Table 1. The main components of the SSO used in this study were α-linoleic acid (25.90%), γ-tocopherol (20.18%), linoleic acid ethyl ester (20.08%) and α-tocopherol (16.54%). The GC–MS chromatogram of the oil is provided in Fig 1.

**Table 1:** Chemical composition of volatiles in sesame seed oil

Peak #	Compounds	Retention time (min)	Percentage content (%)	Molecular formula	Molecular weight
1	10-Bromo undecanoic acid	4.09	0.10	C_11_H_21_BrO_2_	265.19
2	Cholestane-3,7,12,25-tetrol	6.21	0.24	C_27_H_48_O_4_	436.70
3	Benz (a) anthracene -7-carbonitrile	6.30	0.10	C_9_H_11_N	253.30
4	(E,E)-2,4-Decadienal	6.80	1.41	C_10_H_16_O	152.23
5	Phthalic acid, di-(3,5-dimethylphenyl) ester	7.11	0.39	C_24_H_22_O_4_	374.40
6	1,2-Dihydropyrido (3,2,1-kl)phenothiazin-3-one	7.21	0.10	C_15_H_11_NOS	253.32
7	Methyl 2-oxo octadecanoate	7.56	0.10	C_19_H_36_O_3_	312.50
8	1,5-Diphenyl-1H-1,2,4-triazole-3-thiol	7.73	0.10	C_14_H_11_N_3_S	253.32
9	6-Carboxypterin	9.01	0.19	C_7_H_5_N_5_O_3_	207.15
10	1-(1-Adamantyl carbonyl)-4-(2-furoyl) piperazine	9.96	0.05	C_20_H_26_N_2_O_3_	342.40
11	N,N-Dimethyl-1-adamantyl carboxamide	10.26	0.15	C_13_H_21_NO	207.31
12	β-alanyl-L-alanine	10.51	0.05	C_6_H_12_N_2_O_3_	160.17
13	Hexadecanoic acid methyl ester	10.69	0.63	C_18_H_36_O_2_	284.50
14	α-linoleic acid	11.44	25.90	C_18_H_32_O_2_	280.40
15	Linoleic acid ethyl ester	11.51	20.08	C_20_H_36_O_2_	308.50
16	Butyl octadeca-9,12-dienoate	12.62	8.25	C_22_H_40_O_2_	336.60
17	γ-Tocopherol	15.55	20.18	C_28_H_48_O_2_	416.70
18	DL-α-Tocopherol	16.07	16.54	C_29_H_50_O_2_	430.70

### Body and organ weights

After exposing rats to SA alone for 8 days, there was a significant (*P*<0.05) reduction in the body weights of the rats compared to the control group (Table 2). However, treatment of rats with SSO alone or in combination with SA produced significant (*P*<0.05) improvement in the body weights when compared to rats exposed to SA alone. Nevertheless, liver and colon weights remained unaltered in all the groups of rats.

**Table 2:** Effect of SSO on body and organ weight changes in SA-induced toxicity in rats

		Group A	Group B	Group C	Group D
Body weights	**Initial (g)**	207.80±8.66	209.71±4.61	209.00±5.28	206.00±2.09
	**Final (g)**	210.25±7.5	198.83±4.13^a^	219.67±5.65^b^	209.00±3.46^b^
Liver weight (g)		6.37±0.24	6.98±0.32^ns^	7.52±1.46^ns^	6.87±0.86^ns^
Colon weight (g)		1.71±0.39	1.27±0.30 ^ns^	1.18±0.27 ^ns^	1.50±0.27 ^ns^

Superscript ^a^ indicates a significant difference (*P*<0.05) compared with Group A. Superscript ^b^ indicates a significant difference (*P*<0.05) compared with Group B. ns = not significant

### Effect of SSO on liver function enzymes in the serum of rats exposed to SA

Serum activities of liver enzymes are presented in Table 3. In rats intoxicated with SA, the serum activities of ALT and AST were significantly (*P*<0.05) higher than those of control rats, while cotreatment with SSO caused significant (*P*<0.05) reversal of the SA-induced increase in the activities of these enzymes. The serum activity of ALP, however, remained unaltered in all the groups at the end of the treatment period. Notably, administration of SSO alone to normal rats produced no significant changes in AST and ALP levels compared to the control group, while ALT activities were lowered even further than values recorded for the control rats.

**Table 3:** The effect of SSO on the serum activities of ALT, AST and ALP in rats exposed to SA

Parameters	Group A	Group B	Group C	Group D
ALT	28.75±1.25	34.46±0.68^a^	24.94±5.93^b^	23.51±3.15^b^
AST	23.28±0.65	28.21±0.49^a^	24.50±0.33^b^	24.48±0.21^b^
ALP	23.92±7.81	23.46±1.95^ns^	22.90±5.52^ns^	23.00±6.51^ns^

Superscript ^a^ indicates a significant difference (*P*<0.05) compared with Group A. Superscript ^b^ indicates a significant difference (*P*<0.05) compared with Group B. ns = not significant

### Effect of SSO on micronucleus induction in the bone marrow of rats exposed to SA

**Fig. 2:**
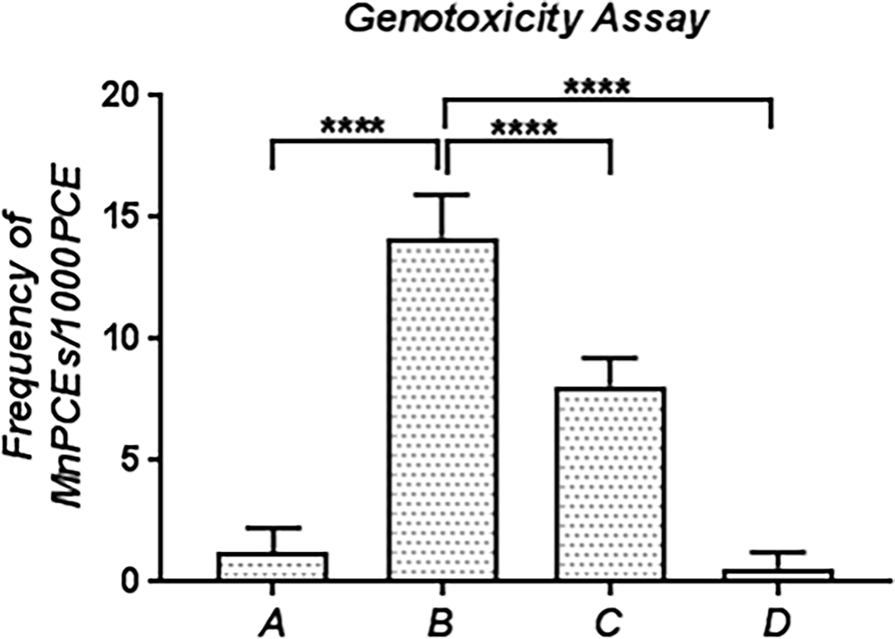
The effect of SSO on micronucleus induction in the bone marrow cells of rats exposed to SA. MnPCEs = Micronucleated polychromatic erythrocytes; PCE = Polychromatic erythrocytes. ****indicates a significant difference at *P*<0.0001

The appearance of micronuclei in the bone marrow cells was evaluated to assess the genotoxic effects of SA. The effect of SSO on the frequency of micronucleated polychrmomatic erythrocytes (MnPCEs) in SA-exposed rats is presented in Fig. 2. The frequency of MnPCEs was significantly increased (*P*<0.0001) in SA-treated rats compared to control rats. However, cotreatment of SA-treated rats with SSO resulted in a significant (*P*<0.0001) decline in the formation of MnPCEs when compared to the rats treated with SA alone. Quite significantly, MnPCEs were almost undetectable in rats treated with SSO alone.

### Effect of SSO on histological changes in the liver and colon of rats exposed to SA

#### H&E

**Fig. 3:**
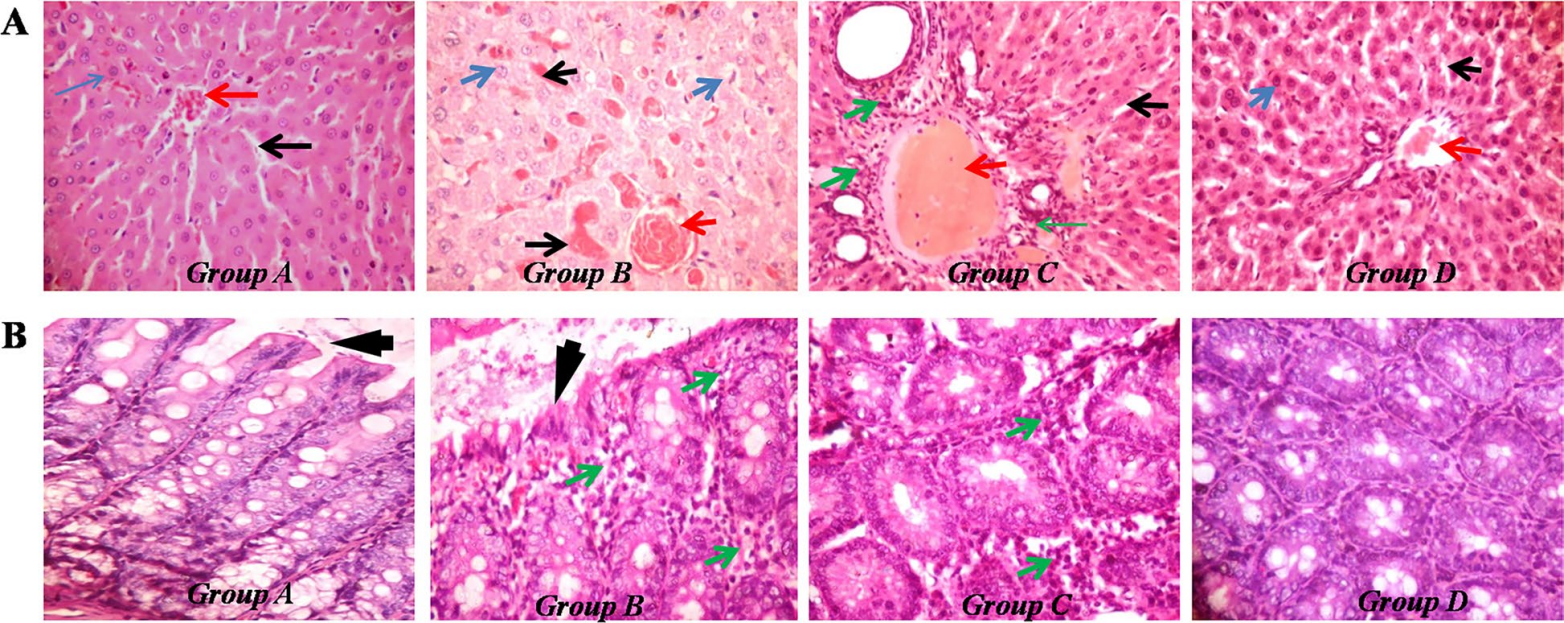
Representative photomicrographs of sections of the liver and colon in rats treated with SA and SSO. **A** H&E-stained histopathological features (100×) of rat livers. Liver tissues of SA-treated rats (Group B) showed necrosis, degeneration of hepatocytes and appearance of apoptotic bodies (blue arrow), congestion of central vein (red arrow) and sinusoids (black arrows). Group C (SA + SSO) showed a lower degree of degeneration of liver tissue, with inflammatory cell infiltration (green arrow), although congestion of the central vein did not show any improvement. Sections from Group A (control) and Group D (SSO only) were well preserved and showed no abnormalities. **B** H&E-stained histopathological features (400×) of the rat colon**.** The colon tissues from Groups A and D showed normal architecture with normal morphology of the epithelium, mucosal and submucosal glands. Group B (SA-treated) showed distortion of the epithelium, with possible erosions (black arrow) and inflammatory cell infiltration (green arrows). Group C (SA + SSO) showed well-preserved epithelium, but a few inflammatory cells were still observed (green arrows)

Liver and colon tissues were stained with hematoxylin and eosin (H&E) to examine abnormalities in the morphology of the tissues. Representative photomicrographs are presented in Fig. 3. Microscopic examination revealed marked destruction of liver tissues in the SA-treated rats, which showed largely unrecognizable morphology. Compared to the control group, the SA-treated rats showed congestion of the central venules and dilated sinusoids, which appeared hemorrhagic, while there was widespread necrosis of the hepatocytes and the appearance of necrotic bodies (Fig. 3A). Treatment of SA-exposed rats with SSO resulted in noticeable alleviation of the pathologies observed with moderately preserved hepatocytes and clear sinusoids, although the central veins still showed congestion and periportal infiltration of inflammatory cells. Liver sections from rats treated with SSO alone showed well-preserved morphology, comparable to those of control rats.

Colon sections from control rats (Group A) and rats treated with SSO alone (Group D) had well-preserved morphology with intact epithelium and prominent glands (Fig. 3B). However, SA-treated rats showed a poorly preserved mucosal epithelial layer, while the lamina propria of the mucosal layer and the submucosal layer showed severe infiltration of inflammatory cells, including lymphocytes and polymorphs. These lesions were, however, ameliorated in rats cotreated with SSO and showed a well-preserved mucosal epithelial layer, although moderate infiltration of inflammatory cells was still observed in the mucosal layer.

#### PAS

**Fig. 4:**
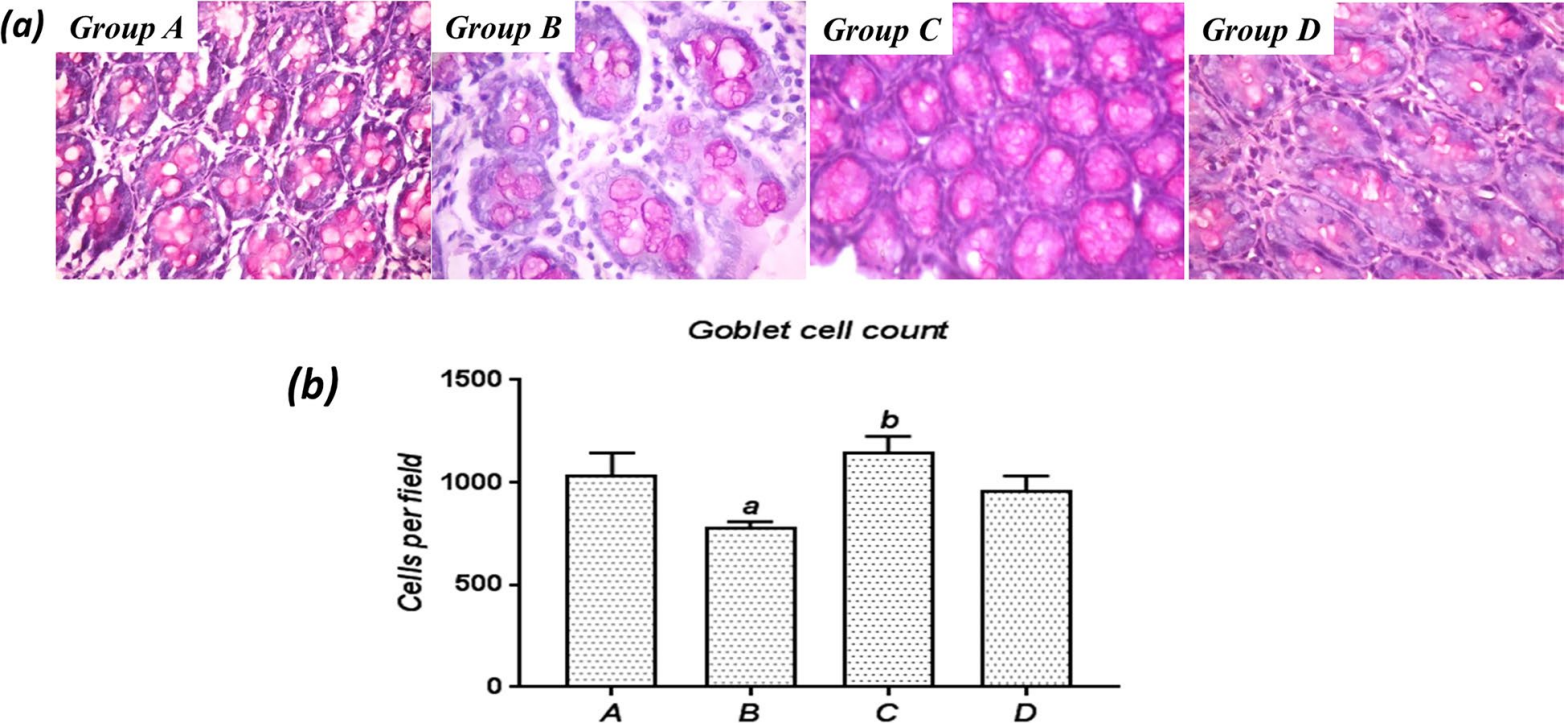
**a** Representative photomicrographs showing mucin expression in colonic sections stained with periodic acid-Schiff (PAS), 400×. Note the pink colored areas of PAS-positive staining indicating mucin production. Group A (control): high glandular and intracellular mucin production; Group B (SA only): moderate glandular mucin production with normal intracellular mucin production; Group C (SA + SSO): abundant glandular mucin production and moderate intracellular mucin production; Group D (SSO only): abundant glandular mucin production with normal intracellular mucin production. **b** Number of goblet cells per field examined. Superscript ^a^ indicates a significant difference (*P*<0.05) compared with Group A. Superscript ^b^ indicates a significant difference (*P*<0.05) compared with Group B

To investigate whether SA was associated with altered mucin production and the modulatory role of SSO, histological slides were stained with PAS to demonstrate unsubstituted α-glycol-rich neutral mucins. Compared to untreated rats, colon sections from SA-treated rats exhibited weak PAS positivity (Fig. 4a), as well as a significantly (*P*<0.05) decreased number of PAS-positive cells per low power field (Fig. 4b), suggesting suppressed glandular mucin production following 8 days of SA exposure. However, treatment with SSO alone or concurrently with SA produced strong PAS staining in the colonic mucosa with significantly (*P*<0.05) increased goblet cell numbers compared with the SA-treated rats.

### Colonic Bcl-2 and CEA immunostaining

#### Bcl-2

**Fig. 5:**
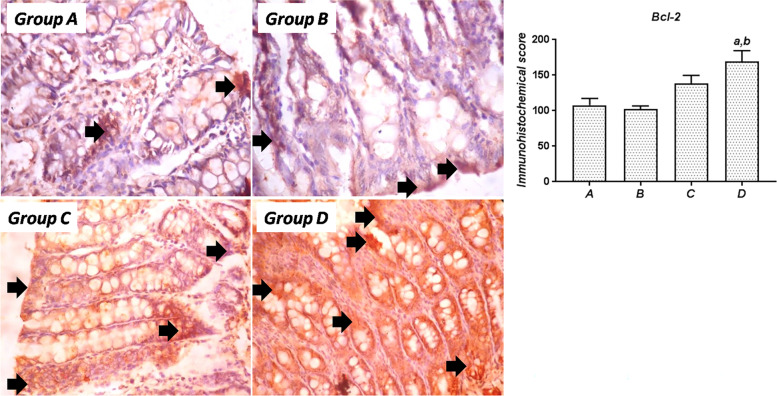
Immunohistochemical expression of Bcl-2 in the colon of rats from the experimental groups. Group A: Control rats showing relatively weak Bcl-2 expression; Group B: SA-treated rats showing colonic Bcl-2 expression similar to that of control rats; Group C: Rats cotreated with SA and SSO showing strong Bcl-2 expression; Group D: SSO-treated rats showing strong Bcl-2 immunostaining. Quantification of immunostaining shows significantly increased Bcl-2 staining in SSO-treated rats compared to control and SA-treated rats. Magnification, 400×

In colonic samples obtained from control rats and SA-treated rats (Groups A and B), moderate levels of Bcl-2 immunoreactivity were detected in virtually all areas of the mucosa, including the crypts and the epithelium. Thus, the intensity and pattern of Bcl-2 expression in the colon samples from SA-treated rats were not different from those of the control rats. In rats treated with SSO alone or concurrently with SA (Groups C and D), the pattern of Bcl-2 immunoreactivity remained the same, also involving the entire mucosa. However, the intensity of Bcl-2 staining in these rats was significantly (*P*<0.05) higher than that in the control and SA-treated rats, indicating a profound upregulation of Bcl-2 by SSO (Fig. 5).

#### CEA

**Fig. 6:**
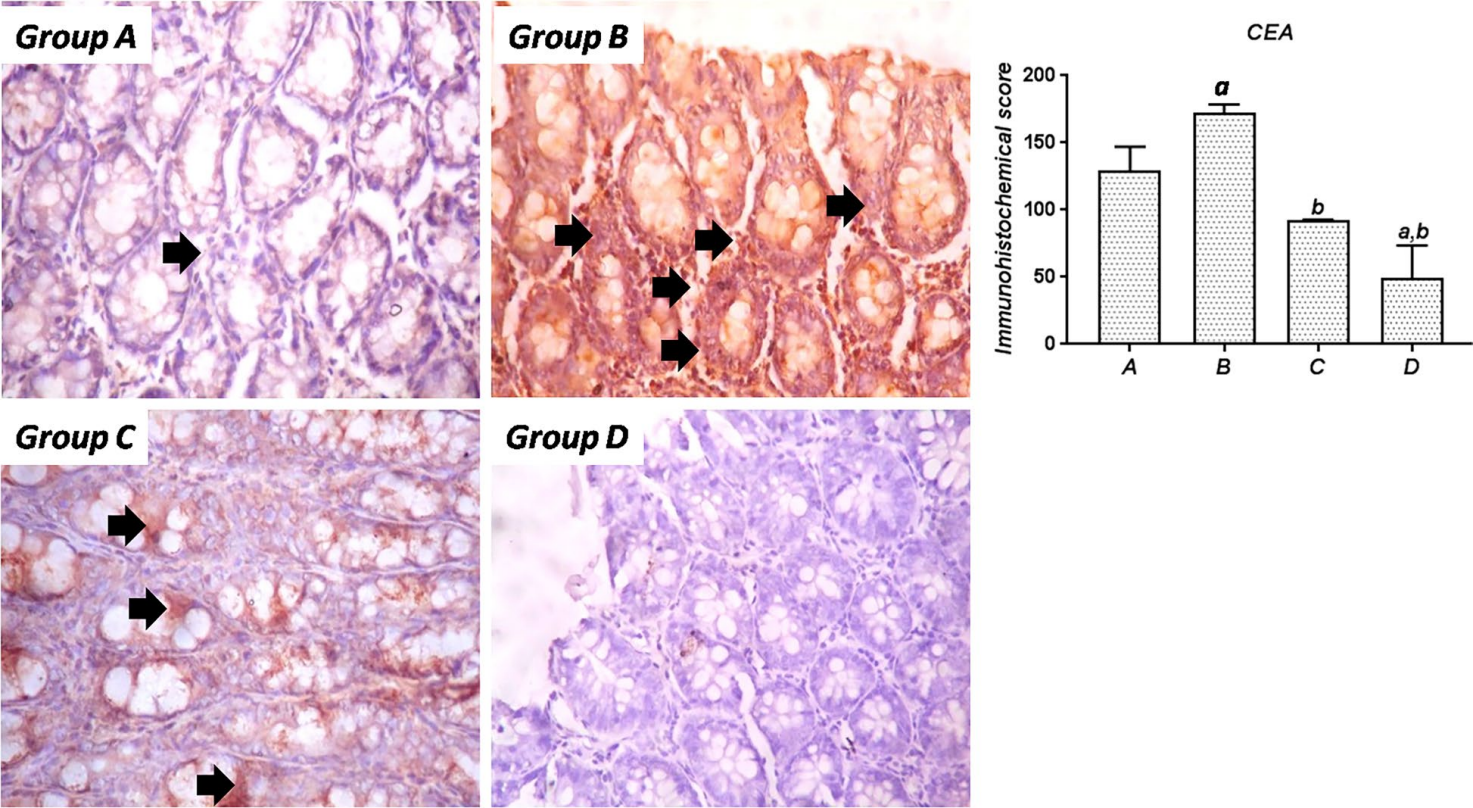
Immunohistochemical expression of CEA in the colon of rats from the experimental groups. Group A: Section from control rats showing normal colon mucosa; Group B: Colon section from SA-treated rats showing intense upregulation of CEA; Group C: Colon sections from rats cotreated with SA and SSO showing reduced expression of CEA compared to rats treated with SA alone; Group D: Sections from SSO-treated rats with no CEA expression. Magnification, 400×

The immunoreactivity of colon samples from the different groups to CEA is depicted in Fig. 6. Rats in the control group (Group A) only showed very mild expression of colonic CEA compared to the SA-treated rats, which showed abundant CEA immunoreactivity across the whole section of the colon. Treatment of SA-exposed rats concurrently with SSO (Group C) resulted in a significant (*P*<0.05) reduction in colonic CEA expression compared to the SA-treated rats, with immunoreactivity confined more toward the crypts. In rats treated with SSO alone (Group D), CEA expression was virtually nonexistent compared to the other groups.

### Colonic MUC-1 and AE1/AE3 immunostaining

#### MUC-1

**Fig. 7:**
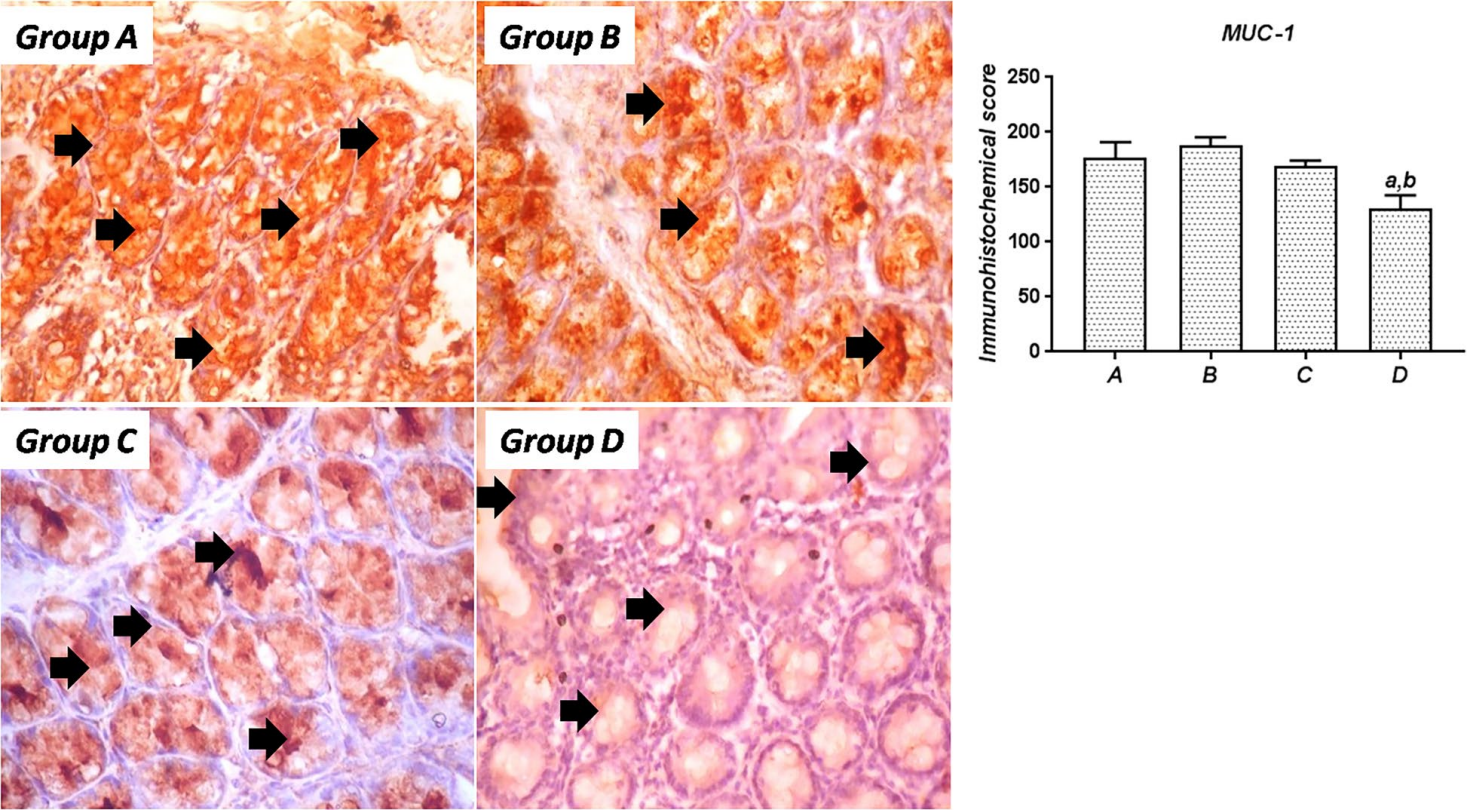
Immunohistochemical expression of MUC-1 in the colon of rats from the experimental groups. Group A: Colonic section from control rats showing intense MUC-1 immunoreactivity; Group B: SA-treated rats with abundant MUC-1 expression, similar to control; Group C: Sections from rats cotreated with SA and SSO still showing abundant MUC-1 expression; Group D: Colon section from SSO-treated rats with significantly reduced MUC-1 expression. Magnification, 400×

The expression of MUC-1 in the colonic mucosa of the experimental rats is depicted in Fig 7. Rats in the normal control group (Group A) showed high expression of MUC-1, and a similar observation was made in rats treated with SA (Group B) following 8 days of exposure. However, treatment with SSO produced a noticeable reduction in MUC-1 expression, although this was not statistically significant upon analysis. In contrast, MUC-1 expression was significantly (*P*<0.05) reduced in rats treated with SSO alone compared to both the control and SA-treated rats.

#### AE1/AE3

**Fig. 8:**
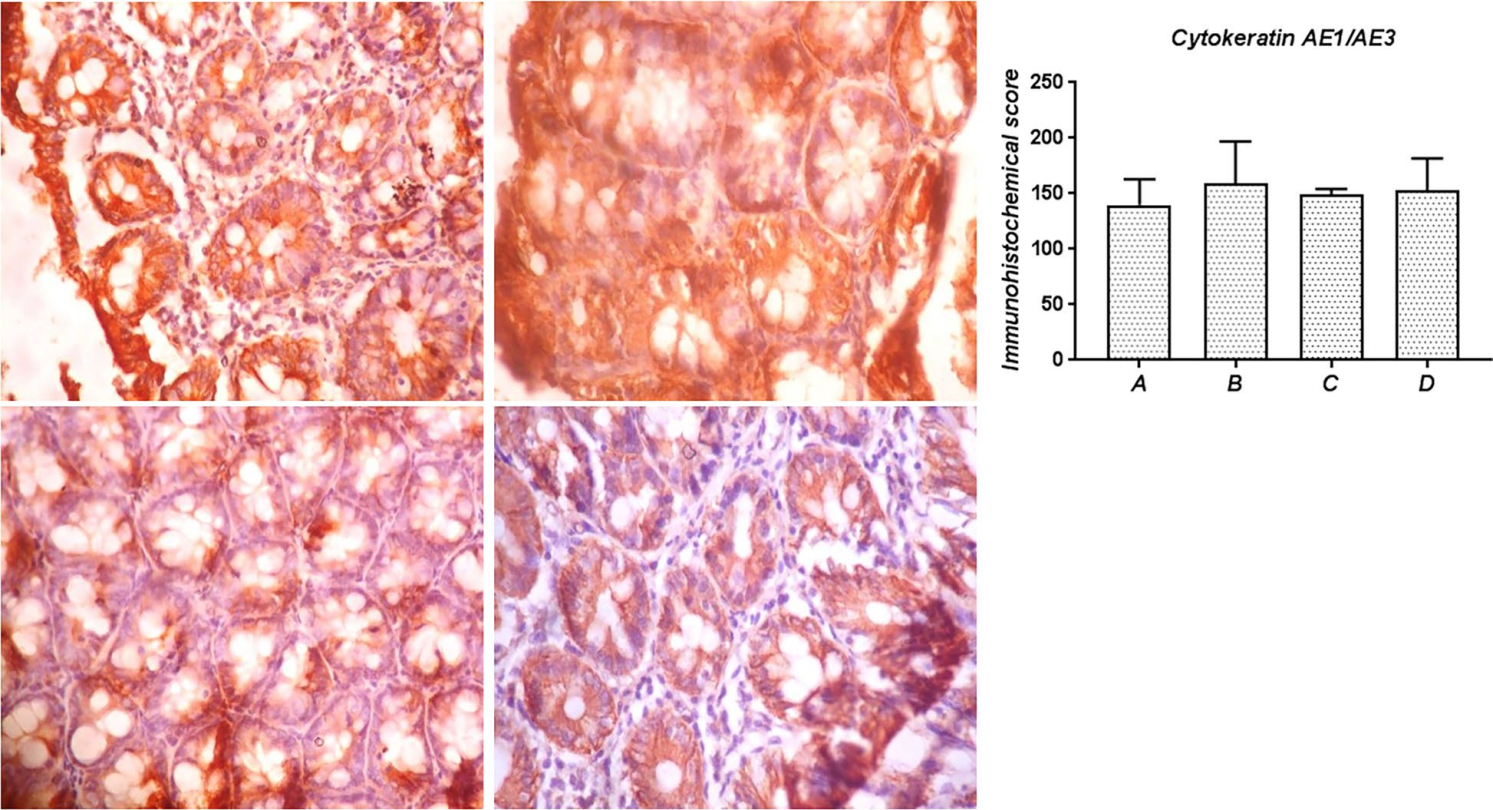
Immunohistochemical expression of cytokeratin AE1/AE3 in the colon of rats from the experimental groups. Group A: Colon sections from control rats with abundant AE1/AE3 expression; Group B: Sections from SA-treated rats also showing abundant AE1/AE3 expression; Group C: Colonic sections in rats cotreated with SA and SSO showing abundant expression of AE1/AE3; Group D: Sections of colon from SSO-treated rats showing abundant AE1/AE3 expression. Magnification, 400×

As presented in Fig. 8, the intensity of expression of cytokeratin AE1/AE3 was abundant in the colon mucosa of all the experimental groups and was not significantly altered in all the groups, irrespective of the treatments. Similarly, the pattern of expression of these proteins was the same across all the groups, with immunoreactivity distributed across the whole mucosa.

## Discussion

Environmental exposure to arsenic compounds continues to be a major issue of public health and concern, as well as a high-priority topic for toxicological research. Acute toxic effects of arsenic poisoning, including gastrointestinal symptoms, bone marrow depression, hepatotoxicity, melanosis, cardiovascular and nervous dysfunctions, are well known to occur following ingestion of arsenic [33]. However, the pathological effects of arsenic exposure in the gastrointestinal tract, especially the colon, are not fully known. Therefore, the present study was directed at investigating the potential protective effect of sesame seed oil on SA-induced hepatotoxicity, bone marrow toxicity and, in greater detail, colonic toxicity by studying the acute responses of some molecular markers of cellular and mucosal injury in the colon, including B-cell lymphoma-2 (Bcl-2), carcinoembryogenic antigen (CEA), mucin glycoprotein 1 (MUC1) and cytokeratins AE1/AE3.

Arsenic is widely accepted as a hepatotoxicant, and accordingly, biochemical assays of serum enzymatic indicators of liver injury in the present study also revealed a significant increase in serum activities of ALT and ALP following 8 days of SA exposure compared with the control group. This finding of arsenic-induced liver injury is corroborated by the report of Yang *et al*. [34] and, as widely reported in other studies, has been attributed to increased generation of reactive oxygen species [6]. Furthermore, we found marked morphological changes, including necrosis and degeneration of hepatocytes with congestion in the central veins and sinusoids, which further confirmed the adverse effects of SA. Other researchers showed similar arsenic-induced lesions, such as degeneration/necrosis, congestion, sinusoidal dilatation and inflammatory cell infiltration, as obtained in the present study [35].

Treatment of rats with SSO significantly alleviated the serum biochemical and histopathological alterations indicative of SA-induced hepatic dysfunction and, in the present study, provided initial indication of the protective potential of the oil. Similarly, as part of its protective activities, SSO treatment caused a reduction in inflammatory cell infiltration into the colonic mucosa while also preserving the epithelial architecture from SA-induced damage. These effects could be attributed to the reported antioxidant and anti-inflammatory activities of SSO [36] and the abundance of bioactive constituents, including α-tocopherol and γ-tocopherol, as well as unsaturated fatty acids, predominantly α-linoleic acid and linoleic acid ester, as revealed by GC–MS analysis of the oil. Previous studies on the chemical composition of sesame oil obtained from different sources have shown that the oil contains large amounts of natural antioxidants, including mono- and polyunsaturated fatty acids as well as vitamin E [37, 38].

Arsenic is known to induce chromosomal aberrations in susceptible cells via induction of oxidative stress during which free radicals attack DNA to produce breakage in chromosomes, especially during cell division. This genotoxic effect of arsenic has been well studied in the bone marrow cells of experimental rats by the demonstration of micronuclei formed in polychromatic erythrocytes [39]. Here, we found that exposure to SA for 8 days induced a significant increase in the formation of micronucleated polychromatic erythrocytes in bone marrow tissues compared to control rats. However, concurrent treatment of rats with SSO resulted in a significant (*P*<0.0001) reduction in the frequency of micronuclei compared to the rats treated with SA only. The results also showed that micronuclei were barely observed in the rats treated with SSO alone, indicating that the oil did not exert any genotoxic action. The anti-clastogenic potential of SSO observed in the present study may be attributed to its reported radical scavenging and antioxidant activities [40].

One distinct feature of the gastrointestinal tract is the possession of goblet cells that synthesize and secrete glycoproteins (mucins), which constitute the mucus that overlies, lubricates and protects the surface epithelium from aggressive factors such as toxins while also stabilizing the presence of some enteric bacteria [41]. There is increasing interest in the role of intestinal mucins in intestinal protection because factors such as exposure to toxic substances and dietary changes tend to affect either the rate of production of mucin or its degradation, thereby affecting the vulnerability of the intestinal mucosa to toxic substances and susceptibility to chronic inflammatory diseases [42]. Thus, an understanding of how chemicals, such as arsenic modulate the mucus barrier is important to developing strategies to combat these diseases. In the present study, there was a profound reduction in the colonic mucus content of rats treated with SA compared to control rats. This was corroborated by marked depletion of goblet cells revealed by PAS staining, which suggests that the lower amounts of mucus in SA-treated rats were likely due to reduced production rather than an increase in mucus degradation.

The mucin family comprises both transmembrane and secreted glycoproteins designated MUC 1 through MUC 21. While secreted mucins, e.g., MUC 2, MUC 5 and MUC 6, form the bulk of the mucus layer overlying the epithelium, the roles of transmembrane mucins, e.g., MUC1, MUC4 and MUC13, are not well defined but are thought to be primarily associated with signaling pathways that are involved in tumorigenesis [43]. Specifically, increased membrane expression of MUC1 is believed to correlate directly with cancer progression [44]. Our findings in the present study indicated that arsenic exposure did not significantly alter the levels of MUC1 in the colonic mucosa, despite the observed depletion of the mucus layer demonstrated with PAS. Therefore, it might be reasonable to suggest that the depletion of the mucus layer observed in SA-treated rats was related to a reduction in secreted mucins rather than transmembrane mucins, such as MUC1. Previous histological observations in ulcerative colitis patients have indeed associated a depletion of goblet cells and mucus secretion with decreased MUC2 synthesis and secretion in the colonic epithelium [45]. Similarly, treatment of rats with dextran sulfate sodium in another study revealed an initial increase in MUC2 and MUC3 expression in the first two days, followed by a rapid decline from the third day of administration [46]. These studies support the suggestion that the decline in mucus secretion in our study was more likely associated with a reduction in mucins other than MUC1.

Interestingly, while SSO administration appeared to stimulate mucus secretion in the colon, it also led to a decline in the expression of MUC1. Previous studies have shown that certain bioactive components of some plants exert gastrointestinal barrier protection by acting as secretagogues stimulating mucus secretion via mechanisms involving the release of endogenous factors such as acetylcholine, prostaglandins, histamine and serotonin [47]. Wu *et al*. [48] reported the ability of the Chinese ‘herbal laxative’, Rhubarb, to stimulate colonic mucus secretion via the aggregation and activation of mast cells with consequent release of histamine, which induces mucus secretion by acting directly on goblet cells. According to Wu and others, these findings were directly correlated with increased MUC2 synthesis and increased proliferation of goblet cells.

Goblet cells normally undergo accelerated exocytosis upon exposure to a mucin secretagogue with acute release of stored mucin granules [49]. Other studies have attributed an increased capacity for mucin secretion to increased goblet cell proliferation and accelerated epithelial turnover [42]. As noted earlier, transmembrane mucins such as MUC1 are more often associated with the development of tumorigenesis and are usually overexpressed in epithelial cancers. It was, therefore, unsurprising to note the strongly positive expression of MUC1 in the colonic mucosa of SA-treated rats. Conversely, the significant reduction in MUC1 expression in rats treated with SSO points to the potential ability of the oil to inhibit the early stages of tumorigenesis via its anti-inflammatory and antioxidant activities [48].

Apoptosis is an active cell death mechanism with distinct biochemical and morphological features. The process is especially important in the gastrointestinal tract for the maintenance of epithelial cell numbers, where tissue homeostasis is maintained by a balance between the rates of apoptosis and mitosis. Very importantly, apoptosis is critical for the elimination of preneoplastic and neoplastic cells [50]. Among several cellular mediators of apoptosis, the BCL-2 family of proteins is considered to contain several important members, prominent among which are the antiapoptotic Bcl-2 and the proapoptotic Bax. Inhibition of apoptosis promotes cell survival, especially following toxic injury, growth factor deprivation or hypoxia, although prolonged inhibition of apoptosis occurring via overexpression of Bcl-2 and other antiapoptotic proteins may also result in hyperplasia and a tendency for malignant transformation [7, 8]. In the present study, Bcl-2 expression in SA-treated rats was maintained at levels similar to those of control rats, suggesting that the rates of apoptosis in the colonic tissues may not have been altered by SA treatment. However, we observed apparent induction of Bcl-2 expression in the colonic tissues of rats treated with SSO either alone or combined with SA, with Bcl-2 levels significantly higher in rats treated with SSO alone.

This finding reinforces recent important arguments about the overall benefit of supplementation of diets with antioxidants or substances such as sesame oil, which possess antioxidant activity. While antioxidants play important roles in the prevention of ROS-induced damage to DNA, proteins and lipids, thereby inhibiting or delaying the initiation and/or promotion of tumorigenesis, their tendency to inhibit apoptosis may also promote the progression of cancer [51, 52]. By implication, administration of dietary sources of antioxidants such as sesame seed oil in the early stages of tumorigenesis may be more beneficial to intercept the action of free radicals, while its prolonged use or its administration at high doses should be avoided due to potential risks of increasing the rate of cell proliferation, especially in cancer patients.

Carcinoembryogenic antigen has been detected mostly in colorectal, breast and lung tumor tissues and is widely used for the diagnosis and prognosis of tumors in cancer patients [53]. However, its expression in the colon of apparently healthy subjects exposed to arsenic has not been studied. Herein, we studied the colonic expression of this protein to understand the acute tissue response to arsenic exposure, as the protein is believed to be associated with the immune response [54]. The results revealed strongly positive immunostaining of CEA in the colonic mucosa of rats treated with SA only compared to control rats, while rats concurrently treated with SSO showed a significant reduction in CEA expression. This result was further strengthened by the very weak immunoreactivity of CEA observed in rats treated with SSOs alone, suggesting that SSOs effectively inhibited immune responses activated by SA in the rat colon. The significance of this finding is that CEA immunoreactivity may be a useful early marker for toxic responses in the colon.

Cytokeratins include several types of intermediate filament proteins of the cytoskeleton expressed in different types of epithelial cells and have been classified into subtypes 1 through 20 [55]. Monoclonal antibodies that detect cytokeratin subtypes are often used in diagnostic immunohistochemistry as markers of differentiation of colonic mucosa cells, as well as certain adenomas and carcinomas of the colon [56, 57]. Cytokeratin AE1/AE3 refers to a cocktail of two different clones of anti-cytokeratin monoclonal antibodies that has a broad spectrum of reactivity against a wide range of high- and low-molecular-weight keratins present in normal and abnormal tissues [58]. The expression of these cytokeratins is frequently organ- or tissue-specific and is therefore used in recognizing epithelial cells of neoplastic origin. The results from the present study show that the colonic epithelium in all the colon samples from the different groups of rats, including controls, was strongly positive for AE1/AE3, indicating that the treatments administered did not specifically alter the constitutive expression of these proteins. Previous *in vitro* studies utilizing continuous exposure of HaCaT cells to low, environmental levels of arsenic for approximately 20 weeks have indicated markedly altered expression of cytokeratin genes at the transcript and protein levels, and this was linked to a transformation of the cells into a malignant phenotype [59]. To the best of our knowledge, the present study is the first attempt to investigate the effect of *in vivo* arsenic exposure on colonic expression of cytokeratin AE1/AE3, although the duration of exposure may be insufficient to observe significant changes in the expression of these proteins.

### Clinical significance of the present study

Arsenic is present everywhere in the human environment. Although arsenic toxicity is often associated with long-term exposure, cases of acute arsenic poisoning have been reported following inadvertent ingestion of insecticides and pesticides and occasionally from attempted suicide. Gastrointestinal and hepatic effects, such as vomiting and diarrhea are mostly observed following oral ingestion and include lesions of toxic hepatitis and hemorrhagic gastroenteritis. In addition, hematopoietic effects, including bone marrow depression, pancytopenia and cytogenetic changes, are commonly found accompanying both acute and chronic arsenic exposure. In a case report by Hasanato *et al*. [60], elevated arsenic concentration in 24-hour urine of a 39-year-old woman was found to result from ingestion of rice contaminated with arsenic. With nonspecific, atypical signs such as diarrhea, headache, insomnia, loss of appetite, abnormal taste, impaired short-term memory and concentration, and absence of skin lesions, it was concluded that signs of acute arsenic exposure can often be insidious and easily overlooked. Therefore, the development of more specific biomarkers of exposure is very important. Arsenic poisoning is often managed with chelation therapy, including oral treatment with dimercaptosuccinic acid, although metal chelators often present with adverse side effects, necessitating the development of natural products and dietary remedies with the potential to ameliorate the toxic effects of arsenic, with little or no risk of adverse effects. Our findings potentially shed new light on the role of putative markers in less-investigated tissues such as the colon.

## Conclusions

The present study demonstrated that SSO administration provided effective protection against SA-induced toxicity in the bone marrow, liver and colon of rats. The protective effects of SSO were manifested as a significant reduction in micronuclei formation in bone marrow cells, reduced serum activities of hepatic enzymes, and preservation of liver and colonic morphology. The protective effects of SSO were likely mediated by its major constituents, including α-linoleic acid and tocopherols. The findings revealed that the cytoprotective activities of SSO in the colon of arsenic-treated rats probably involved a reduction in cellular apoptosis indicated by an upregulation of Bcl-2, along with reduced expression of CEA and MUC-1. The highly significant increase in CEA expression in SA-treated rats suggests that this biomarker could find useful application in the early detection of colonic injury in response to toxicant exposure. Further studies are recommended to clarify the specific roles of the major active components of SSO in the protection against SA-induced adverse events in the liver and intestines.

## Data Availability

The authors confirm that the data supporting the findings of this study are available within the article [and/or] its supplementary materials.
